# Thoracoscopic surgery for bronchobiliary fistula: a case report

**DOI:** 10.1186/s13019-014-0139-z

**Published:** 2014-09-18

**Authors:** Yen-Shou Kuo, Shih-Chun Lee, Hung Chang, Chung-Bao Hsieh, Tsai-Wang Huang

**Affiliations:** Division of Thoracic Surgery, Department of Surgery, Tri-Service General Hospital, National Defense Medical Center, 325, Cheng-Kung Road 2nd section, Taipei, 114 Taiwan; Division of General Surgery, Tri-Service General Hospital, National Defense Medical Center, Taipei, Taiwan

**Keywords:** Bilioptysis, Biliary fistula, Bronchial fistula, Thoracoscopic surgery

## Abstract

The bronchobiliary fistula is an abnormal interconnection between the biliary tract and bronchial trees. It is rare but troublesome. The management of such fistula is a challenge. Surgical intervention is considered after failure of conservative treatment. Here we presented the successful video-assisted thoracoscopic surgery for secondary bronchobiliary fistula in 68-year-old hepatocellular carcinoma patient.

**Electronic supplementary material:**

The online version of this article (doi:10.1186/s13019-014-0139-z) contains supplementary material, which is available to authorized users.

## Background

A bronchobiliary fistula (BBF) is a rare condition that was first reported by Peacock in 1850 [1]. A BBF is an abnormal connection between the biliary tract and bronchial tree. An acquired BBF is generally regarded to be a consequence of a local infection, trauma, biliary tract obstruction, or neoplasm as most are related to hepatic trauma, subphrenic abscess/infection and hepatocellular carcinoma. The presence of bilioptysis and repeated infection could make the diagnosis. However, managing this fistula is a challenge and is often associated with high morbidity and mortality rates. After conducting a literature search, there is surprising very little information regarding thoracoscopic management for an acquired BBF. Here we present our experience with a thoracoscopic intervention for an acquired BBF that, to the best of our knowledge, has not been previously reported.

## Case presentation

**Figure 1:**
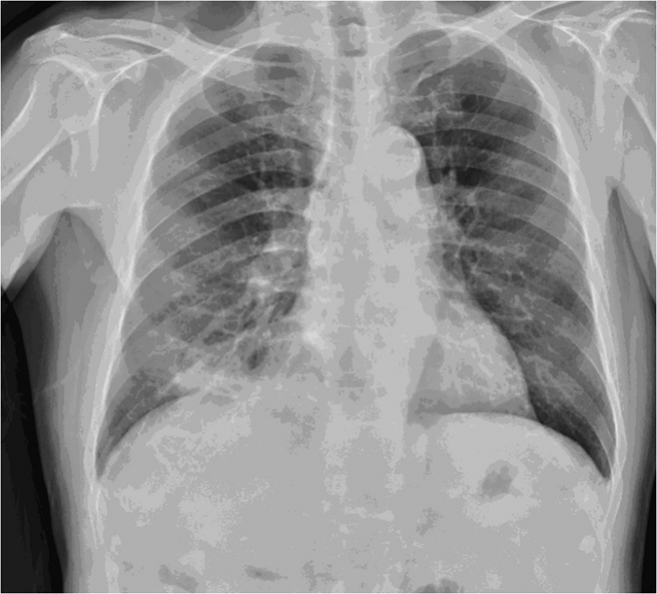
**Shadows are noted in the lower right region of the lungs on a chest X-ray.**

**Figure 2:**
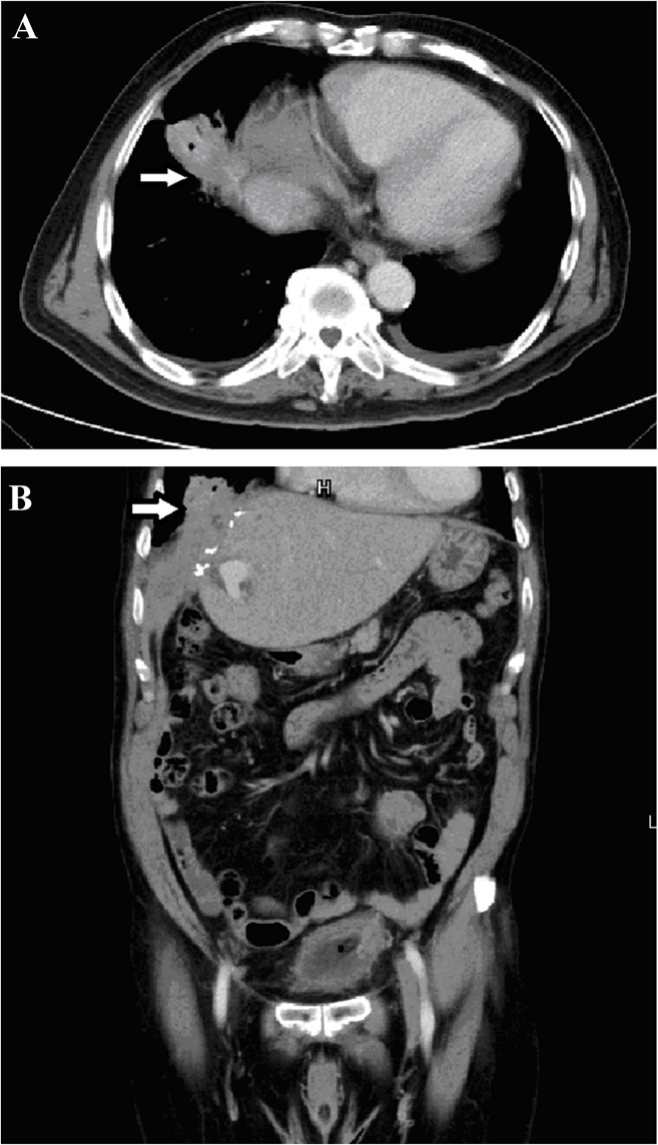
**Computer tomography of abdomen. A:** The axial view showed a lobulated heterogenous mass lesion in the right lower lobe. **B:** The coronal view showed a lobulated heterogeneous lesion in the surgical area over segment 4 of the liver which was adjacent to the diaphragm. Lobulated mass above the diaphragm was also noted.

**Figure 3:**
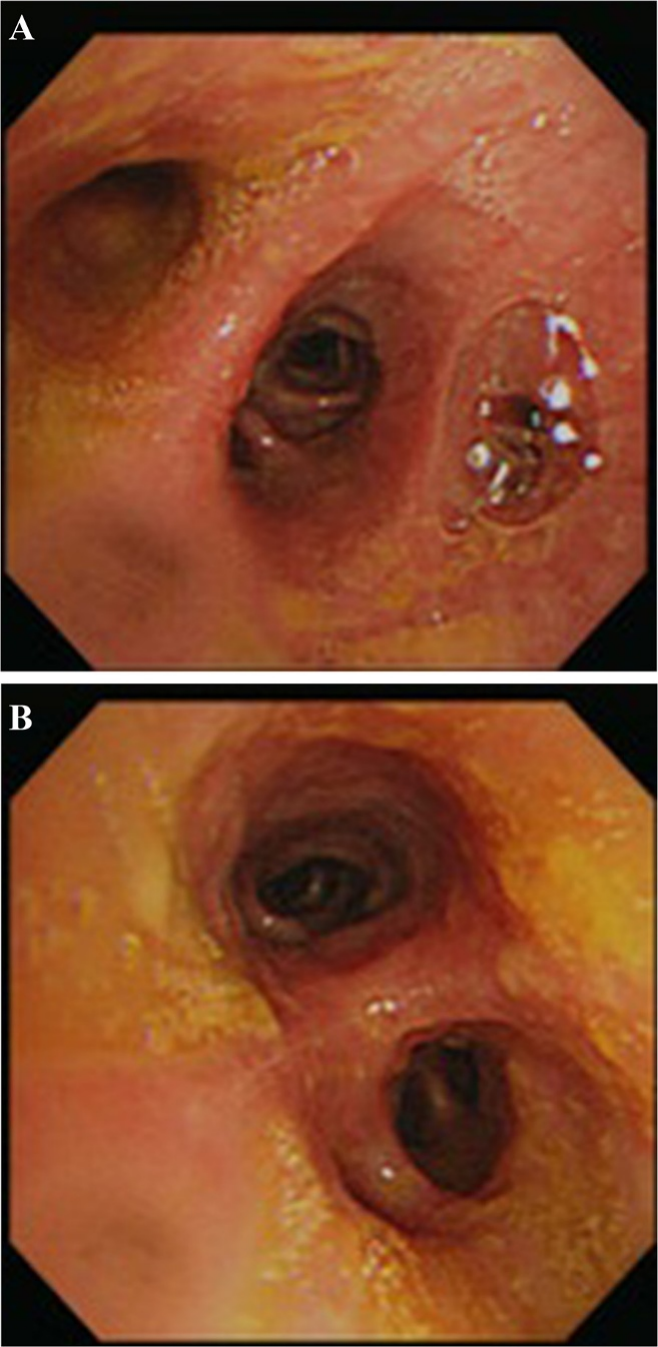
**Flexible bronchoscopy. A:** Yellowish material coating the right lower bronchus. **B:** Yellowish material coating the carina.

A 68-year-old Asian male, nonsmoker was referred to our ward with consolidation in the right lower lung (Figure 1) accompanied by green sputum and bitter saliva. He had been diagnosed with stage I non-B, non-C hepatocellular carcinoma of the liver in segment 4, which was treated with laparotomy and central lobectomy 2 years ago before this admission. Six months later after operation, he underwent percutaneous transhepatic cholangial drainage and endoscopic retrograde cholangiopancreatography (ERCP) with plastic stent implantation because of postoperative strictures of common bile duct. The stent was removed 3 months before the current presentation. At the current admission, physical examination revealed decreased breath sounds in the right lower lung field. Dyspnea was not observed. A reverse L-shaped scar measuring approximately 12 cm in length was noted on the right upper quadrant of the abdomen. There was no rebound tenderness or muscle guarding. The sputum was positive for bile. Chest computed tomography (CT) confirmed consolidation of the right lower lung (Figure 2A&B). Flexible bronchoscopy revealed that the right lower bronchus was coated with bile-like material (Figure 3A&B). These findings led to the suspicion of a BBF. The biochemical study of sputum was positive for bilirubin.

**Figure 4:**
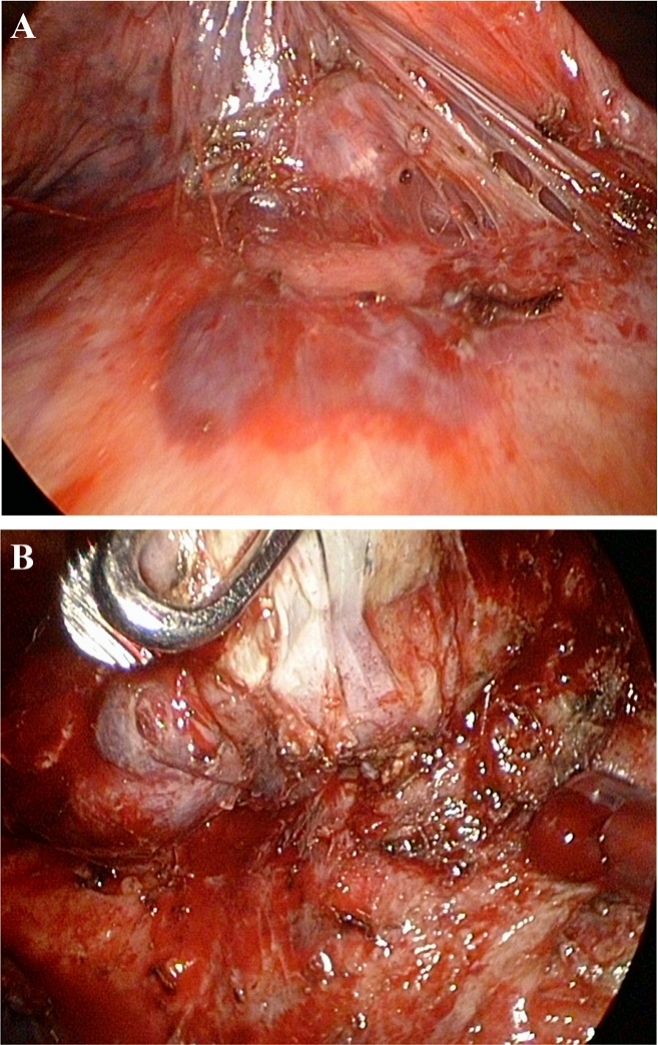
**Thoracosopic finding. A:** Severe adhesion between the diaphragm and lung parenchyma of the right lower lobe. **B:** Pneumolysis is performed and resection of the bronchobiliary fistula (BBF) is achieved.

Under single-lung anesthesia ventilation, we performed a minimally invasive video-assisted thoracic surgery. Using a 10-mm, 30-degree thoracoscope, a 10 mm trocar was placed through the sixth intercostal space in the midaxillary line. A 2 cm incision was placed in the fourth intercostal space in the anterior axillary line. A ring forceps was used through this incision to displace the lung posteriorly and expose the adhesion site between the right lower lung lobe and the diaphragm (Figure 4A&B). After adhesion lysis, disconnection between the lung and diaphragm was performed smoothly using an Endo-GIA.

The patient was discharged 5 days after surgery. There were no signs of bilioptysis with 1 year follow up.

## Discussion

Here we reported a case of an secondary BBF that was clinically suspected and confirmed by contrast-enhanced CT of the abdomen. The optimal treatment for BBF remains controversial. A number of reports have advocated nonsurgical or conservative therapy as an alternative in some cases. As shown by Yilmaz et al. [2], Singh et al. [3], and Ertugrul et al. [4], conservative therapy with biliary and abscess drainage can be effective for BBF associated with a liver abscess, liver hydatidosis after surgical cyst removal, or biliary tract obstruction due to stones or short strictures and for selected cases of post-traumatic BBF. For other cases, surgery is probably the best option.

Liao et al. [5] considered that open surgery should be the first choice when interventional techniques have failed or if a BBF has developed secondary to tumors, biliary obstruction, or trauma. The type of surgery depends on the primary tumor type, BBF location, and involvement of other structures. The following surgical procedures have been performed for a BBF: drainage of a right subphrenic or hepatic abscess, fistula closure, resection of hydatid cysts or a tumor, biliary drainage caused by a T-tube, and bilioenteric anastomoses. For patients with diaphragmatic, pleural, bronchial, or pulmonary damage, diaphragm closure, pleural drainage, decortication, or different pulmonary resections have been performed. There was no mention of thoracoscopic surgery in our review of the literature. In this presenting case, percutaneous hepatic drainage catheter injury or liver abscess is the possible etiology of BPF. It was difficult to differentiate. However, the stent could not resolve the patient's symptoms. Biloma persisted even with the stent . So we decided to perform VATS procedure for this patient.

We performed a minimally invasive video-assisted thoracoscopic resection of a BBF, following which the patient showed an amelioration of bilioptysis and was uneventfully discharged. Compared with previous case reports of surgical treatment by thoracotomy, video-assisted thoracoscopic surgery can be performed safely, with a shorter hospital stay and similar mortality and complication rates. Eryigit et al. [6] described three patients with BBF's that were treated by thoracotomy. The hospital stay for these patients was >10 days. Although the minimally invasive operation could result in less postoperative pain and shorter hospital stay, thoracoscopic surgery for a BBF is complexity and difficulty for surgeons. The repeated infection resulted in severe adhesion of lung. With the advanced surgical technique and equipment, the thoracoscopic procedure should be considered for treating a BBF. We presented successful management experience in BPF via VATS procedure. We thought that it may be possible, but not for all case. VATS management of BBF may be limited to early BBF.

## Conclusion

In conclusion, the present case report suggests that minimally invasive, video-assisted thoracoscopic resection is a safe and effective means for treating a selective BBF.

## Consent

Written informed consent was obtained from the patient for publication of this Case report and any accompanying images. A copy of the written consent is available for review by the Editor-in-Chief of this journal.

## Authors' information

Yen-Shou Kuo

Education

2004 ~ 2009 National Defense Medical Center

Department of Medicine

2000 ~ 2002 Taipei Municipal Jianguo High School

Working Experiences

08/2012 ~ 03/2014 Resident training, Tri-Service General Hospital.

## Authors’ original submitted files for images

Below are the links to the authors’ original submitted files for images. Authors’ original file for figure 1 Authors’ original file for figure 2 Authors’ original file for figure 3 Authors’ original file for figure 4 Authors’ original file for figure 5 Authors’ original file for figure 6 Authors’ original file for figure 7

## Abbreviations

BBF: Bronchobiliary fistula
CT: Computed tomography
