# Erratum: Thoracoscopic surgery for bronchobiliary fistula: a case report

**DOI:** 10.1186/s13019-015-0280-3

**Published:** 2015-07-26

**Authors:** Yen-Shou Kuo, Shih-Chun Lee, Hung Chang, Chung-Bao Hsieh, Tsai-Wang Huang

**Affiliations:** 1 Division of Thoracic Surgery, Tri-Service General Hospital, National Defense Medical Center, Taipei, Taiwan; 2 Division of General Surgery, Tri-Service General Hospital, National Defense Medical Center, Taipei, Taiwan

After publication of the article [1] it was discovered that this manuscript was mistakenly given a duplicated citation number. The correct citation of 9:198 has now been updated in all versions of this manuscript. We apologise for any inconvenience caused by this error.
