# Supplementary figures and images for: Thoracoscopic surgery for bronchobiliary fistula: a case report
Source: J Cardiothorac Surg. 2014 Sep 18;9:139. doi: 10.1186/s13019-014-0139-z (PMC4172870; doi:10.1186/s13019-014-0139-z)

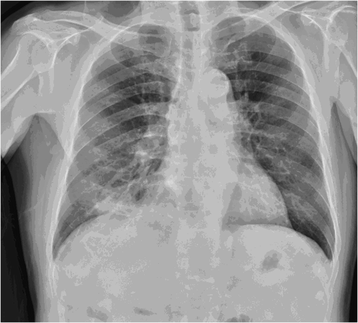

Supplement: Supplementary file 1 — Authors’ original file for figure 1 [file 13019_2014_139_MOESM1_ESM.gif]

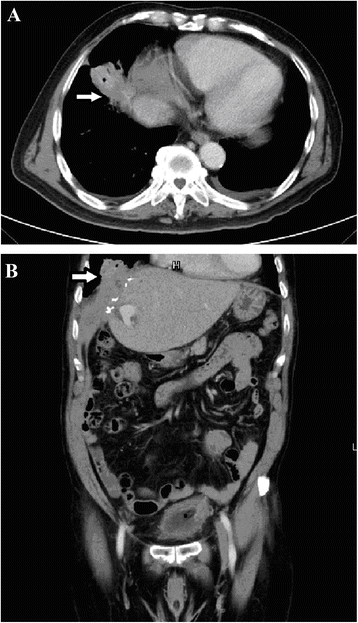

Supplement: Supplementary file 2 — Authors’ original file for figure 2 [file 13019_2014_139_MOESM2_ESM.gif]

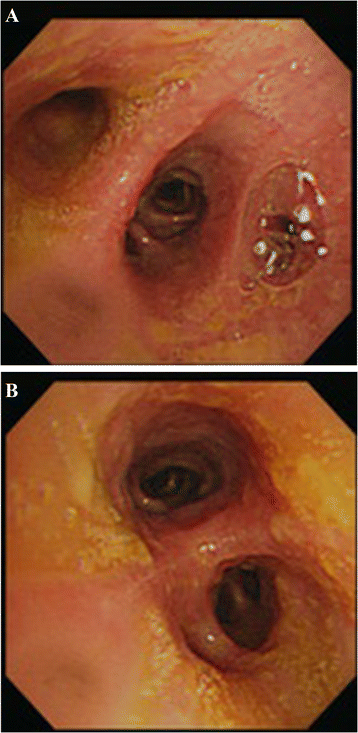

Supplement: Supplementary file 3 — Authors’ original file for figure 3 [file 13019_2014_139_MOESM3_ESM.gif]

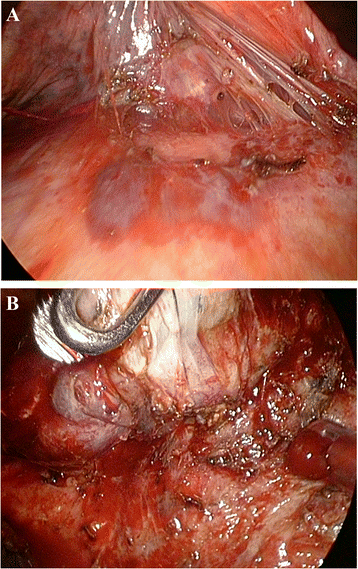

Supplement: Supplementary file 4 — Authors’ original file for figure 4 [file 13019_2014_139_MOESM4_ESM.gif]

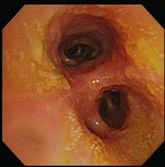

Supplement: Supplementary file 5 — Authors’ original file for figure 5 [file 13019_2014_139_MOESM5_ESM.jpeg]

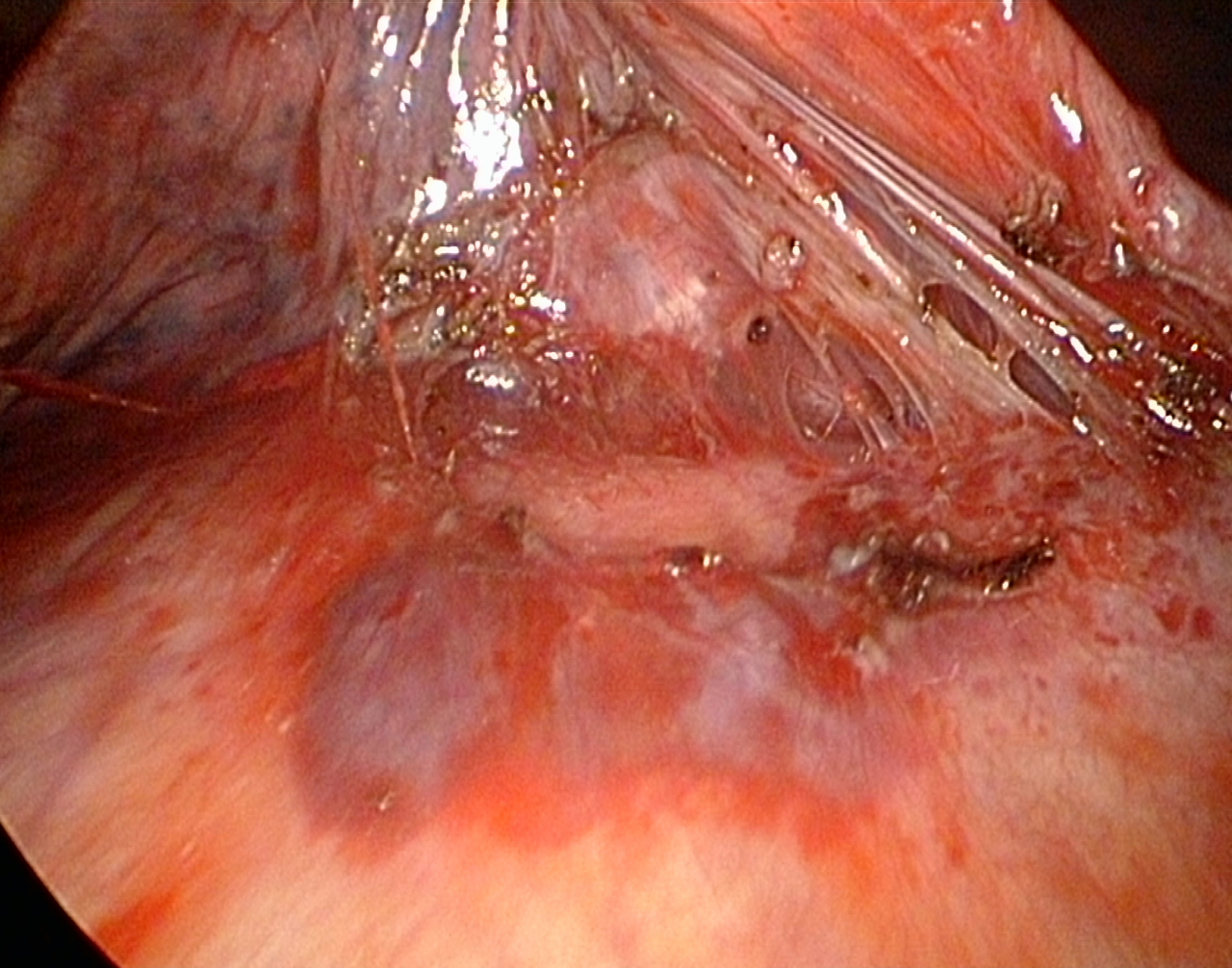

Supplement: Supplementary file 6 — Authors’ original file for figure 6 [file 13019_2014_139_MOESM6_ESM.jpeg]

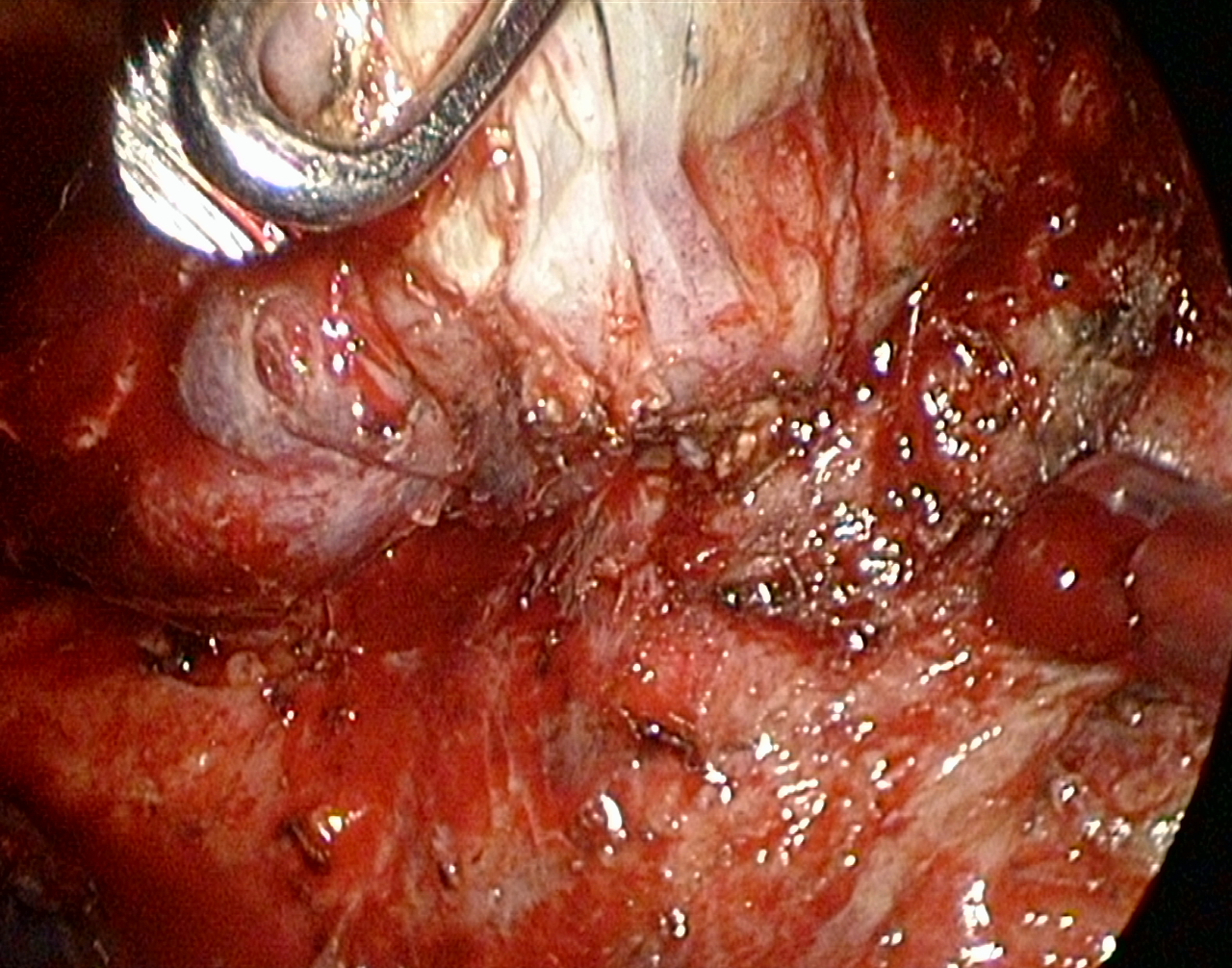

Supplement: Supplementary file 7 — Authors’ original file for figure 7 [file 13019_2014_139_MOESM7_ESM.jpeg]
